# 

**Published:** 1842-11-19

**Authors:** 


### CONTENTS OF No. 47.

Dr. Sale's Case of Obliteration of a part of the intestinal canal, with a rare and unusual formation, - - - - - - - - 737

BIBLIOGRAPHICAL NOTICES: Introductory Lecture to the course of Materia Medica in the University of Pennsylvania. By George B. Wood, M. D., - - - - - - - - - - 737

A System of Human Anatomy, General and Special. By Erasmus Wilson, M. D., Lecturer on Anatomy, London. American Edition, Edited by Paul B. Goddard, A. M., M. D., &c. With one hundred and seventy illustrations by Gilbert. From the Second London Edition. - - - 741

The Medical Student's Guide. By Heber Chase, M. D. - 742

Introductory Lecture to the Course of Medical Institutes in the University of Pennsylvania. Delivered November 4, 1842. By Samuel Jackson, M. D., - - - - - - - 742

ANALECTA: M. Dumas on the Chemistry of Organized Beings, - - 745

Can the future state of the Milk be predicted during Pregnancy? - - - - - - - - - - - - 748

Dr. Blieding on Transfusion of the Blood of a Goat into the Veins of a Man, - - - - - - - - - - 748

M. Piorry on the Efficacy of Injections of Infusion of Cubebs in Vaginitis, - - - - - - - - - - - 749

M. Vautier's Case of Recovery from Poisoning with Corrosive Sublimate, - - - - - - - - - 749

Poisoning by Cyder containing Lead in Solution, - - 749

Dr. Jules Guerin, on the Sanatory condition of Paris during the first six months of 1842, - - - - - - - 750

Case of Poisoning by the Binoxalate of Potash, - - - 751

Icterus, - - - - - - - - - - 751

M. Orfila on the Absorption of Chemical Substances into the Blood, &c., - - - - - - - - - - 752

